# Single-shot wavelength-multiplexed phase microscopy under Gabor regime in a regular microscope embodiment

**DOI:** 10.1038/s41598-023-31300-9

**Published:** 2023-03-14

**Authors:** Vicente Micó, Mikołaj Rogalski, José Ángel Picazo-Bueno, Maciej Trusiak

**Affiliations:** 1 Departamento de Óptica y Optometría y Ciencias de la Visión, Universidad de Valencia, C/Doctor Moliner 50, 46100 Burjassot, Spain; 2 Institute of Micromechanics and Photonics, Warsaw University of Technology, 8 Sw. A. Boboli St., 02‑525 Warsaw, Poland

**Keywords:** Optical imaging, Interference microscopy, Imaging and sensing

## Abstract

Phase imaging microscopy under Gabor regime has been recently reported as an extremely simple, low cost and compact way to update a standard bright-field microscope with coherent sensing capabilities. By inserting coherent illumination in the microscope embodiment and producing a small defocus distance of the sample at the input plane, the digital sensor records an in-line Gabor hologram of the target sample, which is then numerically post-processed to finally achieve the sample’s quantitative phase information. However, the retrieved phase distribution is affected by the two well-known drawbacks when dealing with Gabor’s regime, that is, coherent noise and twin image disturbances. Here, we present a single-shot technique based on wavelength multiplexing for mitigating these two effects. A multi-illumination laser source (including 3 diode lasers) illuminates the sample and a color digital sensor (conventional RGB color camera) is used to record the wavelength-multiplexed Gabor hologram in a single exposure. The technique is completed by presenting a novel algorithm based on a modified Gerchberg–Saxton kernel to finally retrieve an enhanced quantitative phase image of the sample, enhanced in terms of coherent noise removal and twin image minimization. Experimental validations are performed in a regular Olympus BX-60 upright microscope using a 20X 0.46NA objective lens and considering static (resolution test targets) and dynamic (living spermatozoa) phase samples.

## Introduction

Quantitative phase imaging (QPI) is a microscopy discipline aimed to quantify the phase delays happening when light passes through a sample having a spatially variant density distribution^1,2^. Although not being the unique option^3^, digital holographic microscopy (DHM) is doubtlessly the most common technique to achieve QPI, particularly in biological research^4,5^ and industrial applications^6,7^.

Building a simple arrangement is of particular significance in DHM since it provides a compact and cost-effective solution in a comprehensive and easy-to-use way for QPI. Thus, DHM has been proposed using a single interferometric element such as a thick glass plate^8^, a wedge prism^9^, a Fresnel’s biprism^10^, a Lloyd’s mirror^11^, a beam splitter cube^12^ or a diffraction grating^13^, just to cite some examples. Also following in this uncomplicated and compact way to produce QPI, several attempts have been dedicated to convert—with minimal modifications—a regular white light microscope into a microscope with coherence sensing capabilities. Some proposals successfully adapt an external add-on module to the exit port of a regular microscope such as, for instance, modules based on wavefront sensing^14^, Michelson-based layouts^15,16^, lateral shearing interferometers^17,18^, transport of intensity equation (TIE) algorithms^19,20^, diffraction phase microscopy^21^, beam splitter interferometer^22^ or purely numerical kernel^23^.

Going deeper into this simplicity path, probably the easiest way to provide holographic recording is by using the Gabor’s principle of holography^24^ by incorporating a coherent illumination source in a regular microscope embodiment and by introducing a small defocus at the sample plane. The recorded defocused image can be considered as a digital Gabor in-line hologram, where the magnification is not coming from the geometrical projection of the sample at the recording plane—as in the classical lensless geometry—but introduced by the microscope embodiment itself. Imaging is then achieved by digital refocus to the best image plane using well-known numerical propagation algorithms^25^. Maybe the strongest limitation of this methodology is the restriction imposed by the Gabor’s regime, that is, the sample must be weakly diffractive or, in other words, the amount of light blocked/diffracted by the sample should be a small fraction in comparison with the one passing without being perturbed by the sample^26,27^. Nevertheless, there is a wide range of biological samples (single cell analysis, thin structured samples, sparse biosamples, etc.) matching the Gabor’s condition, thus opening a huge potential for the application of this methodology.

Surprisingly, this extremely simple way to produce QPI in microscopy is relatively new^28–31^. Mandula et al. derived phase from defocus^28^ as a simple and compact phase imaging microscope for long-term observation of non-absorbing biological samples as well as its combination with fluorescence microscopy in a single dual-mode imaging platform using a standard bright-field objective^29^. A similar concept for combining fluorescence with phase microscopy was reported by de Kernier et al.^30^. And Micó et al. recently reported on an extremely simple, low cost and compact way to update a standard bright-field microscope with coherent sensing capabilities^31^. In their work^31^, the authors characterized the best axial defocusing to be applied at the sample plane when considering a 20× microscope lens to retrieve full quantitative phase information after the numerical processing stage based on angular spectrum (AS) algorithm. But the main drawbacks in that configuration from a QPI point of view are the same that the presented ones in Gabor’s holography concept: the coherent noise (interference patterns coming from back reflections, coherent artefacts, non-uniformities, speckles, etc.) and the twin image problem that introduce phase disturbances in the retrieved QPI values as a direct consequence of using coherent illumination in an in-line holographic arrangement.

To mitigate all those problems, we present here a wavelength multiplexing approach where 3 wavelength-coded digital holograms are recorded in a single snapshot of a color camera, thus allowing fast events analysis coming from living samples. RGB illumination (using 3 fiber coupled laser diodes) is used to transmit in parallel 3 Gabor holograms and a color digital camera records the multiplexed hologram, from which the 3 wavelength-coded in-line holograms can be extracted. Once separated, the information included within each color-coded channel is numerically processed using a novel algorithm based on a modified Gerchberg–Saxton (G–S)^32^ kernel aided with object plane constraints^33^ to provide improved QPI of the sample, improved in terms of coherent noise removal and twin image minimization.

Similar approaches based on wavelength multiplexing have been proposed in the field of lensless imaging^34–45^. Noom et al. presented a quantitative phase contrast lensless holographic microscope first under sequential illumination/recording^34^ and later with high-speed capabilities^35^. Sanz et al. also reported on a novel concept of a compact, cost-effective and field-portable lensless microscope^36^ based on wavelength multiplexing and a fast and robust algorithm for twin image minimization and noise reduction^37^. They also extended such concept to a lensless imaging platform with different resolutions/magnifications^38^ and considering 4 multiplexing channels^39^. Kazemzadeh et al. proposed the use of pulsed illumination to even expand up to 5 channels and developed a multispectral lens-free microscope for biological specimens^40^. Liu et al.^41^ and Guo et al.^42^ reported on the use of a rotating filter wheel as key concept to provide multiple wavelength illuminations onto the sample in the field of lensless imaging for improving autofocusing^41^ and for noise reduction^42^. Luo et al. proposed a wavelength scanning method for pixel superresolution^43^. Liu et al. recently proposed the use of field of view (FOV) multiplexing for allowing single-shot operational principle in multi-illumination lensless imaging^44^. And Zuo et al. demonstrated lensless quantitative phase microscopy and diffraction tomography based on a compact on-chip platform based on multi-wavelength phase retrieval and multi-angle illumination diffraction tomography^45^.

However, the concept of wavelength multiplexing for image quality improvement working with regular microscopes has not been previously reported to the best of our knowledge. Closely related to the proposed concept is the work proposed by Waller et al. to retrieve phase information based on a transport of intensity (TIE) algorithm, where the axial defocus needed between images is provided by the intrinsic chromatic aberration of the microscope lenses^46^. They used broadband illumination and conventional RGB color camera for providing 3 slightly defocused images (in-focus, and under and over focus) corresponding with the 3 color camera channels and those 3 intensity images were the inputs of the TIE algorithm. Although aimed in the same direction (improve QPI in a single-shot by reducing coherent noise), Waller et al. work is conceptually different to our proposed method. Moreover, it is penalized by the low-frequency noise presented in TIE-based phase reconstructions and it depends on the amount of chromatic aberration introduced by the objective lens, something that must be somehow calibrated before applying TIE recovery.

## Experimental methodology

**Figure 1:**
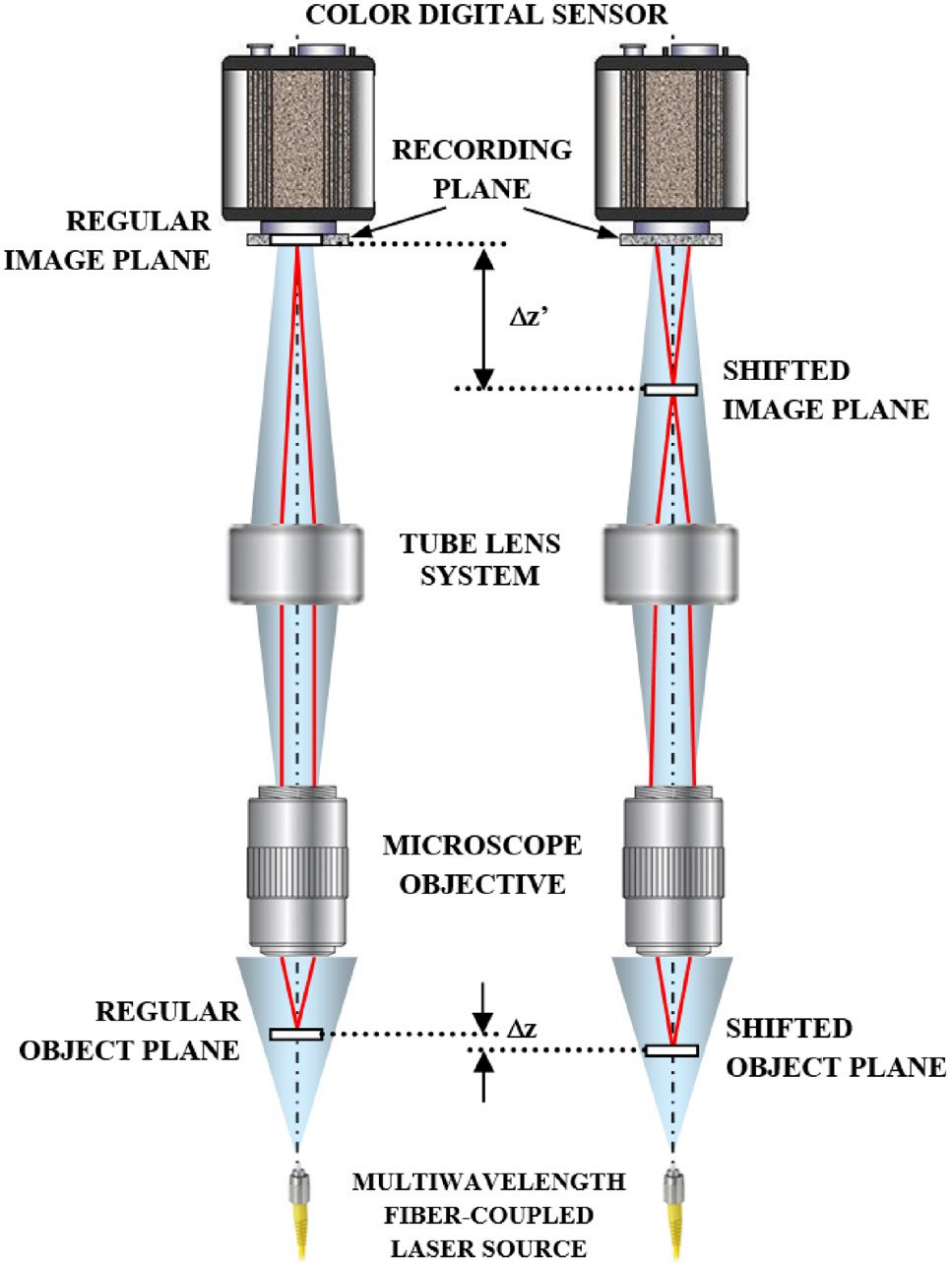
Optical scheme of the proposed approach. The sample is slightly shifted (Δz) from its regular object plane position (left vertical drawing) allowing to shift the conjugated image plane (Δz′) for the digital recording of an in-line hologram (right vertical drawing).

Figure 1 includes a scheme of the experimental layout where the proposed approach has been implemented and validated. It represents a regular upright microscope embodiment (Olympus BX-60 with UMPlanFl 20× 0.46NA objective) where a fiber coupled laser source (Blue Sky Research, SpectraTec 4 STEC4 405/450/532/635 nm) is externally inserted to provide coherent illumination in the system. Essentially, the arrangement is the same as in Ref.^31^ but here we use multi-wavelength simultaneous illumination (450/532/635 nm) instead of a single emitter (as used in Ref.^31^) to provide the 3 color-coded holograms in parallel and a color camera (Ximea USB3 MQ042CG-CM, CMOS sensor type, 2048 × 2048 pixels, 5.5 μm pixel pitch, 90 fps) instead of a monochrome one (as in Ref.^31^) for decoding the 3 transmitted in-line holograms from a single snapshot. In the proposed setup, the defocus distance Δz (see Fig. 1) is one of the key factors and must be properly defined. This value was set experimentally in Ref.^31^ by analyzing 2 different metrics: resolution of the reconstructed USAF test target images and standard deviation (SD) of a background (object free) area. Since we are using here the same layout as in Ref.^31^, the same experimental conditions prevail so the defocusing distance is set to Δz = 90 μm, meaning that the sample is moved away 90 μm from the typical objective working distance. For setups with different parameters (including objective’s NA and magnification, camera characteristics and focal lengths of tube lens), different Δz values will be optimal. Up to the best of our knowledge, no theoretical solution has been proposed for setting optimal Δz distance as a function of the microscope parameters. Therefore, for different arrangements, we propose to repeat Δz calibration process described in Ref.^31^.

**Figure 2:**
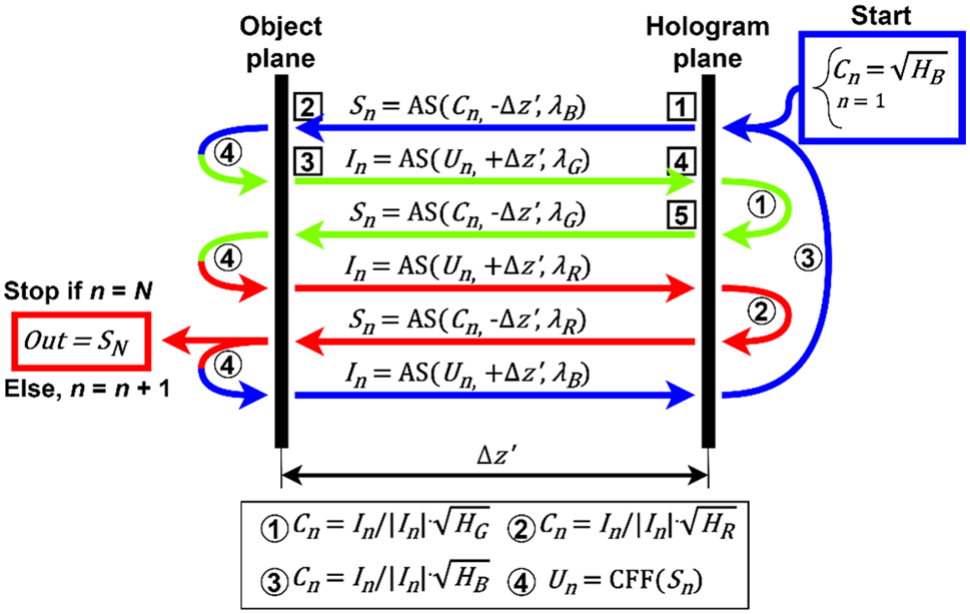
Diagram of the proposed algorithm processing path. Hi represents the hologram recorded with i (R, G or B) wavelength illuminations. Numbers from 1 to 5 in square frames correspond to images in Fig. 3 marked with the same numbers.

**Figure 3:**
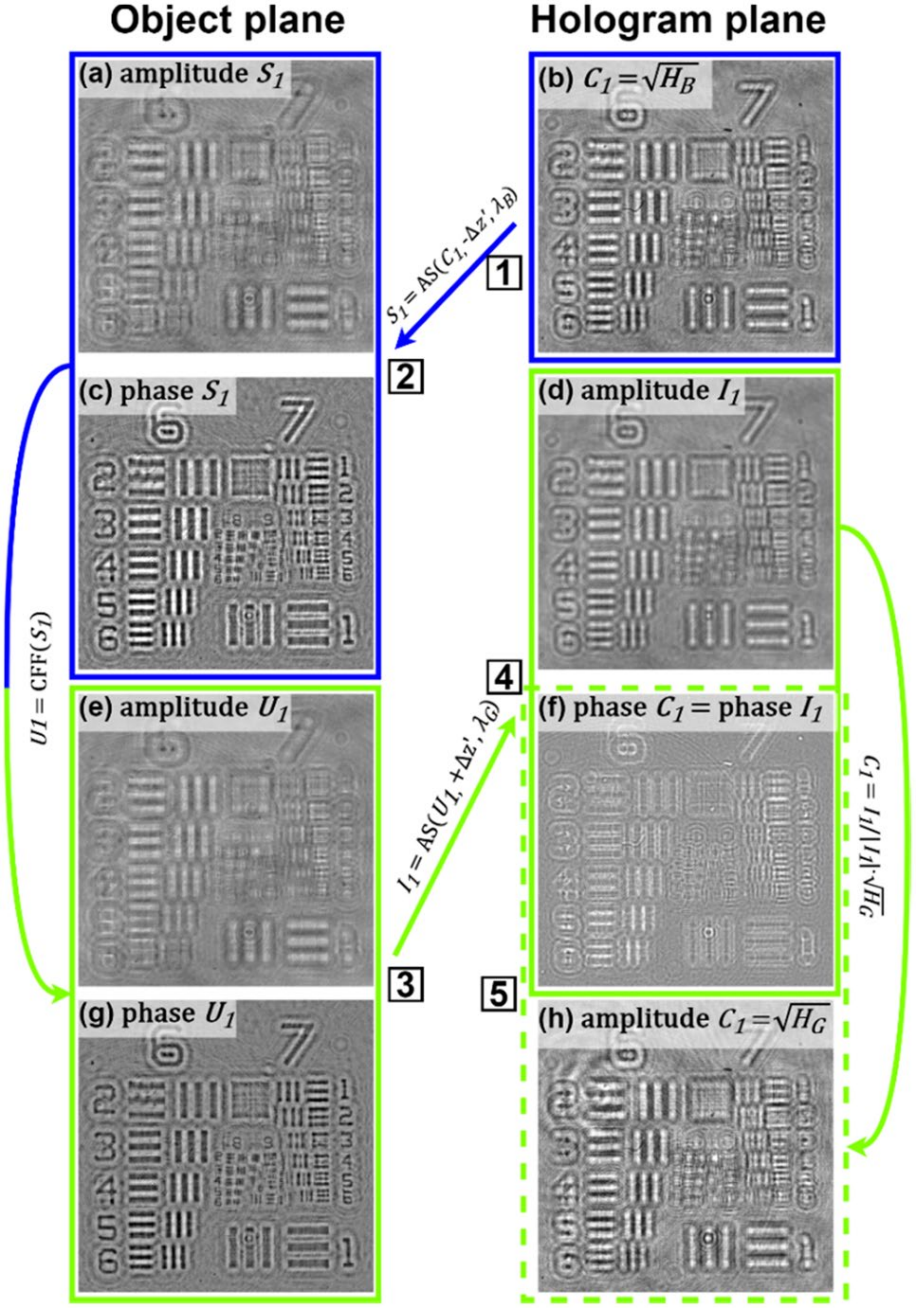
First 4 steps of the proposed algorithm when considering an USAF phase resolution test target. Numbers from 1 to 5 in square frames correspond to parts of the algorithm in the diagram of Fig. 2 marked with the same numbers.

**Figure 4:**
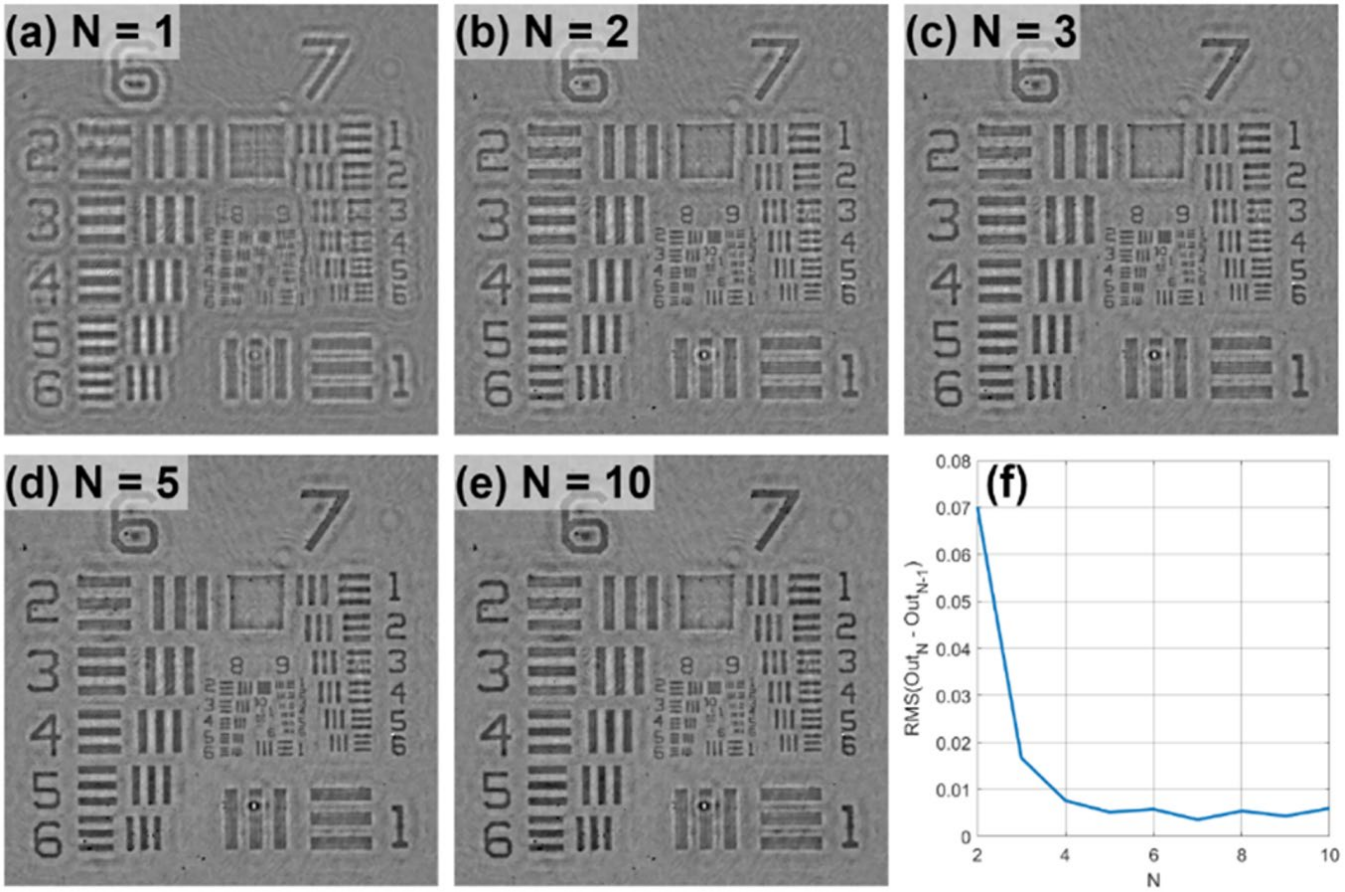
(**a**)–(**e**) Reconstruction of USAF phase test target for a different number of iterations (N). (**f**) RMS of the difference between N and N − 1 iteration results.

Figure 2 shows the diagram of our iterative algorithm processing patch and Fig. 3 presents the propagated optical field during first 4 steps of the algorithm when considering an USAF phase resolution test target. In the first step of the first iteration (n = 1), the optical field at the hologram plane (C_1_) is assumed to be equal to the square root of the blue wavelength hologram (H_B_)—Fig. 3b. Then, it is backpropagated a distance − Δz′ to the object plane with the angular spectrum (AS)^47^ method and considering the blue wavelength illumination (λ_B_)—Fig. 3a and c. Notice that Δz′ will be the same for each wavelength when considering achromatic systems. After that, both real and imaginary parts of the optical field (S_1_) are processed with a novel complex field filtration (CFF) algorithm, which is partially based on a halo removal method presented in Ref.^42^. CFF algorithm for the real part of S_1_ (Re{S_1_}) is described with equations below:

1 $$J_{blr} \left( r \right) = {\text{Re}} \left\{ {S_{1} \left( r \right)} \right\} * w\left( r \right)$$

2 $$\begin{array}{*{20}l} {J_{flt} \left( r \right) = {\text{Re}} \left\{ {S_{1} \left( r \right)} \right\},} \hfill & {\quad if\;{\text{Re}} \left\{ {S_{1} \left( r \right)} \right\} \ge J_{blr} \left( r \right)} \hfill \\ {J_{flt} \left( r \right) = \frac{{{\text{Re}} \left\{ {S_{1} \left( r \right)} \right\} + J_{blr} \left( r \right)}}{2},} \hfill & {\quad if\;{\text{Re}} \left\{ {S_{1} \left( r \right)} \right\} < J_{blr} \left( r \right)} \hfill \\ \end{array}$$

where r stands for spatial coordinates in object plane, w is a gaussian kernel (standard deviation equal 60 and filter dimensions equal 241 × 241 pixels), * stands for convolution operation, J_blr_ is a blurred image and J_flt_ is a filtered image. It is worth to underline that the conditions in Eq. (2) are checked for each pixel separately. The imaginary part of S_1_ is filtered similarly as the real one (with the same gaussian kernel). The resulting optical field after CFF algorithm (U_1_)—Fig. 3eand g—is then propagated to the hologram plane at + Δz′ distance considering in this case the green wavelength (λ_G_)—Fig. 3d and f. At the hologram plane, the optical field (I_1_) is updated by replacing its amplitude part by the square root of the hologram recorded with green illumination (H_G_), while its phase factor remains unchanged – Fig. 3f and h, defining an updated C_1_. Then, this updated C_1_ is further processed, according to the diagram presented in Fig. 2 and considering G (and later R) wavelength illumination. First (or each) iteration finishes with S_n_ backpropagated to the object plane considering the R wavelength illumination (bottom-left part of the diagram in Fig. 2). The algorithm is computed for the user-specified number of iterations (N). Figure 4 shows the exemplary USAF phase test target reconstructions with different number of iterations and Fig. 4f shows the plot of the root mean square (RMS) value of the difference between phase reconstruction in N and N − 1 iteration. As can be observed, after several first iterations, there is a significant improvement in the reconstruction resolute, but after that (since around 5th iteration), the algorithm converges and there is not much difference between the results. Therefore, we usually set N = 5 as it is sufficient to obtain good quality results, without unnecessary increasing the reconstruction time.

## Results

**Figure 5:**
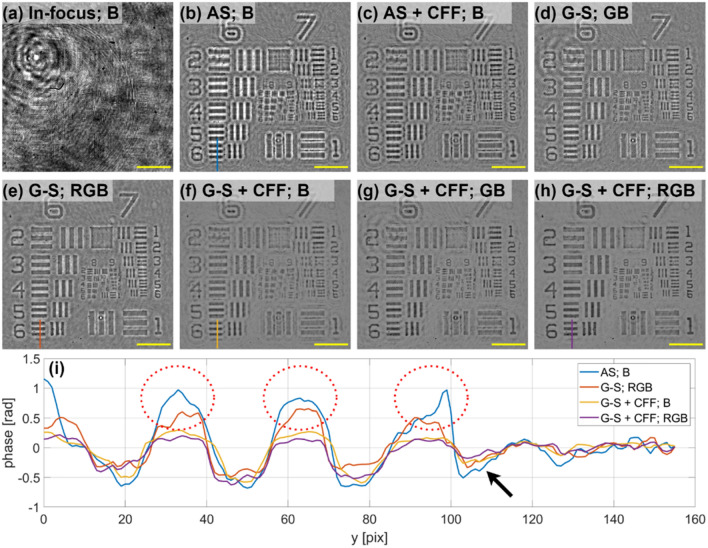
Experimental comparison of different phase reconstruction methods for the case of an USAF phase test target. (**a**) In-focus intensity image considering the blue illumination, and its phase reconstructions from defocused images with: (**b**) AS backpropagation, (**c**) AS backpropagation result filtered with CFF (**d**) GB G–S, (**e**) RGB G–S, (**f**) B G–S + CFF, (**g**) GB G–S + CFF, and (**h**) RGB G–S + CFF methods. (**i**) Cross-sections through the group 6 element 6 of the reconstructed phases. Phase values for (**b**)–(**h**) are displayed in [− 1,1] rad range and the yellow scale bar is 50 μm long.

Figure 5 presents the exemplary reconstructions of the USAF phase resolution test target, comparing various methods. Figure 5a shows the intensity distribution in the in-focus position for the blue wavelength. As the imaged target is transparent, registered image is of very low contrast. Figure 5b presents the phase result of a blue hologram backpropagated with the AS method to the object plane and Fig. 5c shows the AS result filtered with CFF algorithm. Figure 5d and e show the G–S reconstructions without CFF considering 2 (GB) and 3 (RGB) simultaneous wavelengths. G–S reconstructions with CFF for 1 (B), 2 (GB) and 3 (RGB) wavelengths are shown in Fig. 5f–h respectively. Comparing simple AS backpropagation with G–S algorithm, the twin image effect is significantly reduced for G–S, especially for the 3 simultaneous wavelengths case. However, it is still observable, similarly to a “halo” effect (white pixels in Fig. 5d,e). After applying CFF, this “halo-like” effect is almost completely extinguished. Comparing the G–S + CFF reconstructions with single and multi-wavelength cases, single-wavelength case, Fig. 5f, contain significantly higher number of twin-image artifacts (“fake” negative values in the image background) than 2 and 3 wavelength cases, Fig. 5g and h.

Comparing 2 and 3 wavelengths, RGB result seems to be noticeably more robust in terms of coherent noise artifacts removal (compare top left part of FOV in Fig. 5e,f). Given the fact that recording 3 holograms (RGB) has the same level of complexity as recording 2 holograms (GB) with a RGB camera, the 3-wavelength reconstruction provides better outcomes. Cross-sections through Element 6 of Group 6 (E6-G6) of the chosen reconstructed phases are included in Fig. 5i. In the no-CFF reconstructions, some “fake” positive phase values can be observed (marked with red dashed ellipses) in object-free regions, which are only visible on a small scale in CFF cross-sections. When comparing G–S + CFF reconstruction with single and three illuminations, again “fake” negative phase values are observable for single-wavelength case [marked with black arrow in Fig. 5i].

**Figure 6:**
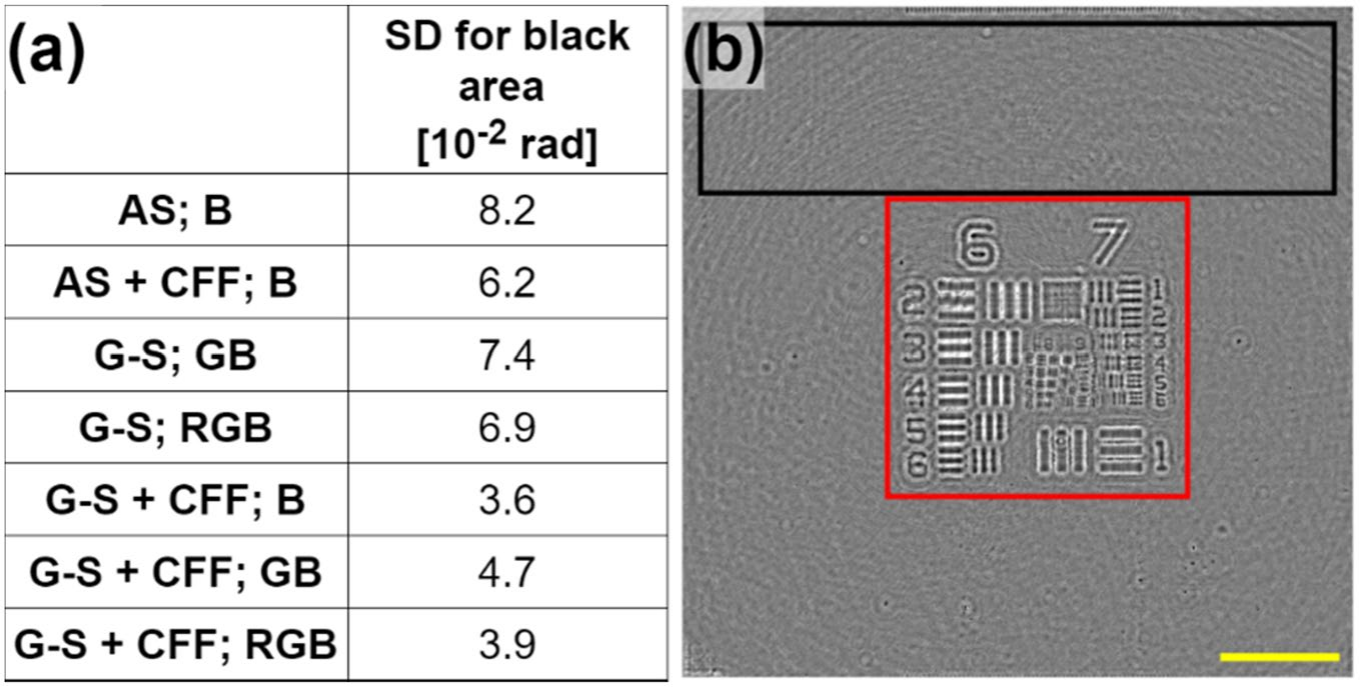
(**a**) Comparison of the background SD values from the reconstructions provided by the different methods. (**b**) Full FOV retrieved phase image with B AS where the black/red rectangles mean the area for SD calculation and the region of interest showed in Fig. 5, respectively. Yellow scale bar is 100 μm long.

Figure 6 includes a standard deviation (SD) analysis of the background for all the compared reconstructions. Figure 6a presents all the SD background values in a table while Fig. 6b shows the region (black rectangle) where the SD values are computed and the full FOV phase image (AS backpropagated with B wavelength illumination) from which the region of interest (red rectangle) is included in Fig. 5. As expected, there is a SD reduction when including additional wavelengths and also with CFF algorithm application as consequence of the averaging, yielding in a reduction factor slightly above 2 when passing from traditional 1 wavelength reconstruction to the proposed method. The only exception is the smaller SD value for G–S + CFF reconstruction with single-wavelength illumination compared to multi-wavelength illumination cases. This is due to the high coherent noise present in G illumination hologram. Nevertheless, despite that fact, RGB reconstruction achieves only slightly larger SD than B reconstruction, which means that this G coherent noise is minimized by the proposed algorithm.

Aside of the validation performed with the static phase target and included in previous figures, maybe the most appealing feature of the proposed method relates to its capability to work in a single shot. To demonstrate this feature, we have conducted experiments using a live human sperm sample. The sperm cells have approximately a head length and width of 4 and 5 μm, respectively, a total length of 45 μm and a tail width below 1 μm. The sample is placed in a counting chamber having a depth of 20 μm (Proiser R + D, model ISASD4C20) allowing free swimming of the cells. No pre-filtering nor pre-preparation (centrifugation, dilution, re-suspension, etc.) is applied, so the sample contains a lot of additional seminal particles. Movies are recorded for 4 s at an acquisition rate of 90 fps to study the dynamic motions of the sperm cells.

**Figure 7:**
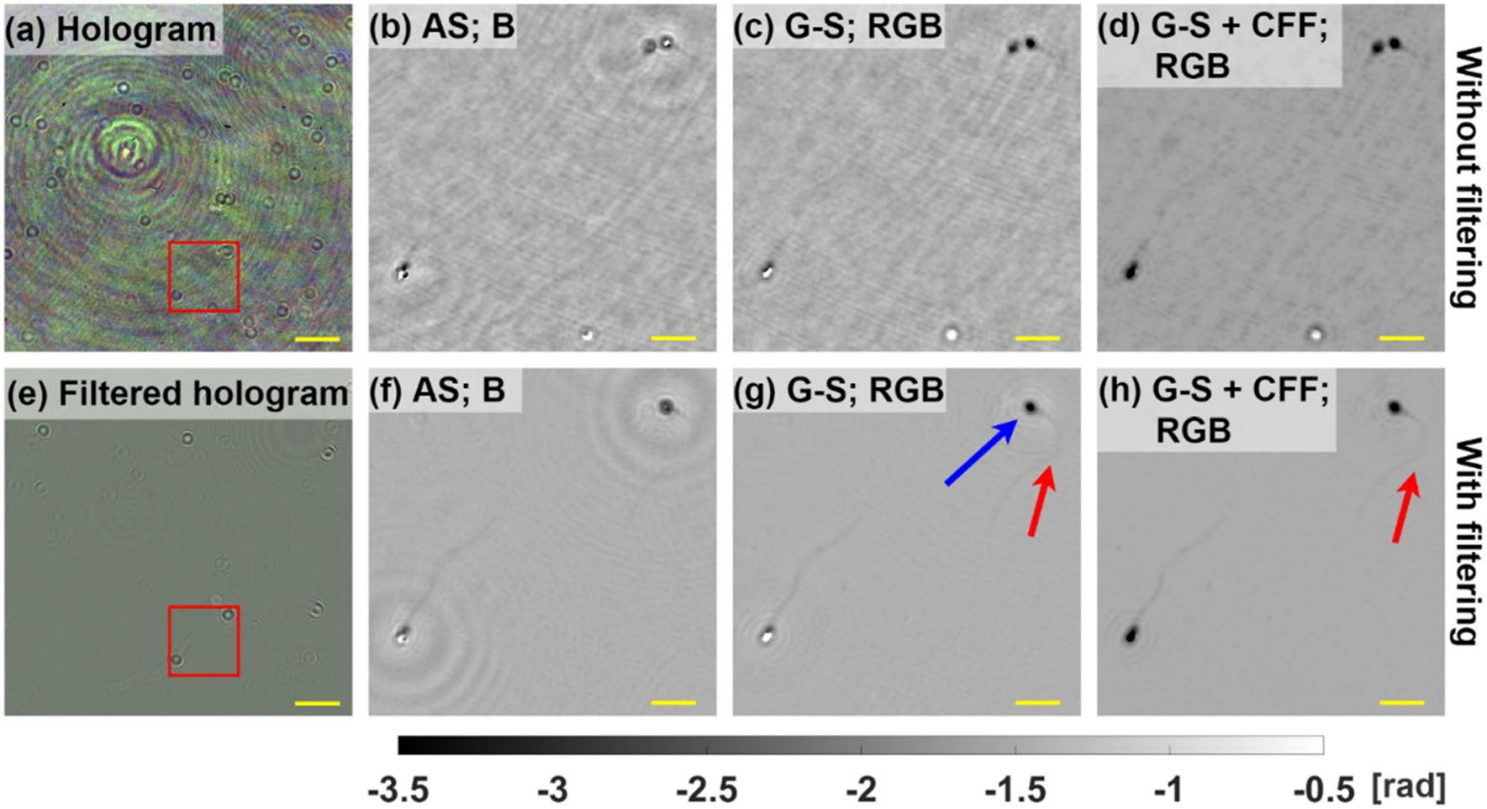
Experimental validation for the case of living sperm cells. Multiplexed hologram without (**a**) and with (**e**) filtration, respectively. Reconstructed phases for holograms without (**b**)–(**d**) and with (**f**)–(**h**) filtration, respectively. Yellow scale bar is 50 μm in (**a**), (**b**) and 10 μm in (**b**)–(**d**), (**f**)–(**h**).

Figure 7 presents the first frames of the obtained movies including the multiplexed recorded hologram—Fig. 7a, the multiplexed recorded hologram after filtering (subtracted mean recorded frame) to remove all static cells and debris—Fig. 7e, and the reconstructed phases for single wavelength backpropagation—Figs. 6f and 7b—RGB G–S without CFF—Figs. 6g and 7c—and with CFF—Fig. 7d and h. Results are presented for both no-filtration—Fig. 7b–d—and with static objects filtration—Fig. 7f–h—cases. For better clarity, phase values are unwrapped, as cells bodies have phase values below − π and the phase reconstructions are shown only for areas marked with red rectangles in Fig. 7a and e. Full FOV reconstructions for filtration free and with static objects filtration cases are presented in Visualization 1 and Visualization 2, respectively. The best results are obtained for G–S algorithm with filtration, where the spermatozoid tail may be observed (marked with a red arrow in Fig. 7g,h) For no-filtration case the spermatozoids tails are not observed, probably due to coherent noise coming from static objects. Additionally, again the smallest twin image is observed for aiding the G–S reconstruction with CFF algorithm, what results in minimizing “halo-like” effect (marked with a blue arrow in Fig. 7g) and avoiding unwrapping errors (bottom-left spermatozoid in Fig. 7f and g.

As proven above, the proposed algorithm can significantly outperform both classical G–S and AS methods in terms of quality of the reconstructed phase. However, this is achieved at a cost of increased computational complexity and therefore, increased computational time. Table 1 presents typical reconstruction times of a 2040 × 2040 pixels hologram on a low-cost laptop (Intel i7, 2.8 GHz CPU and Nvidia GeForce GTX 1060 GPU) for different algorithms. GPU processing was used to optimize algorithms execution time. Proposed algorithm achieved around 15 times longer execution time than the classical AS method. However, despite that fact, below 5 s computation time for 2040 × 2040 holograms should still be enough for most of the applications and should not be too troublesome for the users.

**Table 1:** Computation time of the different algorithms. G–S times are given for 5 iterations.

Algorithm	AS	CFF	G–S; GB	G–S; RGB	G–S + CFF; B	G–S + CFF; GB	G–S + CFF; RGB
Time (s)	0.3	0.65	1.53	2.1	1.54	2.98	4.41

## Discussion and conclusions

Along this manuscript, we have presented a step forward to improve phase imaging microscopy in an upright commercially available microscope, which has been updated with coherent sensing capabilities using the simplest way one can imagine to allow holographic imaging. This is nowadays a remarkably interesting topic because it expands the use of regular microscopes for the analysis of biological samples without the need to manipulate (staining, fixing, etc.) them. Thus, it takes all the advantages provided by actual microscopes concerning image quality and stability with coherent sensing capabilities coming from digital in-line holographic microscopy. Moreover, the low cost of these type of approaches contribute to the democratization of science by allowing to perform phase imaging in biological experiments to those laboratories with constrained budget.

Through the presented images, we have experimentally shown how coherent noise and twin image are mitigated as consequence of the averaging, G–S algorithm and CFF implementation, to finally achieve an output image with improved quality. Part of this improvement is as consequence of the averaging of the 3 images during the numerical methodology but it is also coming from the CFF algorithm itself which can be understood as a way to blur out all the features that have higher amplitude (“negative absorption”) and phase (smaller refractive index) values than the averaged neighboring background area while remaining unchanged all features with lower values. The phase resolution test target case clearly shows image quality improvement (twin image minimization, halo-like mitigation, contrast improvement and background SD reduction). And the living sperm cells experiment shows a real biological application of the proposed approach with the same improvements in phase imaging as in the USAF target case. Here, the phase imaging enhancement allows the visualization of additional parts (full tail) of the cells, which can serve for improving the morphological analysis of the cells as well as having a better information for their 3D tracking.

As in any Gabor’s implementation scheme, the main limitation concerns with the restriction imposed to the target sample (weak diffraction assumption). However, in biology and biomedicine there are plenty of cases where biosamples can be treated as weakly diffractive samples, thus satisfying the Gabor’s condition and being perfect candidates to be imaged with the proposed methodology, which allows in-vivo imaging without modifying the sample environment. Moreover, Gabor’s layout is commonly known by its simplicity, cost-effectiveness, compactness and aberration-free properties. But high NA values are difficult to be achieved on both classical but opposite arrangements in lensfree imaging^48,49^ due to both geometrical distortion and the mandatory compromise between the illumination pinhole diameter, the illumination wavelength, and the need to obtain a reasonable light efficiency^48^ as well as because of the geometrical constraints imposed by the pixel size of the detector^49^. The inclusion of a microscope objective for optically magnifying the sample while recording a defocused diffraction pattern^31^ allows to easily achieve high NA (defined by microscope objective) at the cost of a reduced FOV and the need to control aberrations. Nevertheless, this is not a significant issue in our system probably because of the in-line principle of Gabor’s holography where reference and object beams travel together the same optical path and are affected by the same lenses. Moreover, since modern microscope objectives are quite well aberration balanced for the entire visible spectrum, they do not introduce any significant aberration (distortion, spherical, chromatic, etc.) that could separately affect each color-coded channel image.

Our proposed algorithm assumes ideal separation between camera spectral channels (S_i_, being i = R, G, B) where only a single wavelength (λ_j_, being j = R, G, B) takes contribution on each channel, that is, S_i_ = δ_ij_ · f(λ_j_) being δ_ij_ the Kronecker delta function. However, the presence of crosstalks on each RGB camera channel coming from the two additional wavelengths can influence in the final image quality reconstruction. Although we have not noticed any significant problem concerning this fact because the RGB selected wavelengths are close to the maximum spectral sensitivity of their corresponding camera channels, additional procedures can be defined for the case that crosstalks will be a problem. Thus, on one hand, the crosstalks can be easily removed by subtraction a set of previously recorded images using independent wavelengths^37^ or using more complex procedures involving the definition of the wavelength detector response matrix to correct each channel reading^36,39^. Anyway, these calibration procedures must be done once and the result must be applied to each recorded frame as preliminary digital preparation of the data set before entering into the proposed workflow algorithm.

In summary, we have presented single-shot wavelength-multiplexed phase microscopy implemented in a regular microscope embodiment with minimal modifications for improving phase imaging under Gabor’s regime. Validation is included for a 20X microscope objective, but it is extendable to any other lens by only defining the proper defocusing distance. Just as an application example, we have included the case of living sperm cells in a counting chamber. However, the potentiality of the proposed approach is far beyond that and it can be applied to a long list of biological cases such as, for instance, long-term observation events, including cell division and apoptosis, single cell examinations, cell to cell interactions and imaging flow cytometry.

## Supplementary Information

Supplementary Legends.

Supplementary Video 1.

Supplementary Video 2.

## Data Availability

Data underlying the results presented in this paper are not publicly available at this time but may be obtained from the authors (maciej.trusiak@pw.edu.pl) upon reasonable request.

## Abbreviations

QPI: Quantitative phase imaging
DHM: Digital holographic microscopy
TIE: Transport of intensity equation
AS: Angular spectrum
RGB: Red–green–blue
G–S: Gerchberg–Saxton
FOV: Field of view
USAF: United State Air Force
CFF: Complex filed filtration
SD: Standard deviation
