# Supplementary material for: Single-shot wavelength-multiplexed phase microscopy under Gabor regime in a regular microscope embodiment
Source: Sci Rep. 2023 Mar 14;13:4257. doi: 10.1038/s41598-023-31300-9 (PMC10015059; doi:10.1038/s41598-023-31300-9)
Supplement: Supplementary file 1 — Supplementary Legends. [file 41598_2023_31300_MOESM1_ESM.docx]

**Supplementary file information:**

Visualization 1 (4.7 MB, mpg file):

Related figure: Figure 6.

Legend description: Full FOV reconstruction considering no filtration (direct hologram recording) for single wavelength (left), G-S RGB (central) and G-S RGB with CFF (right).

Visualization 2 (3.4 MB, mpg file):

Related figure: Figure 6.

Legend description: Full FOV reconstructions considering static object filtration for single wavelength (left), G-S RGB (central) and G-S RGB with CFF (right).
